# Biases in Volumetric Versus Surface Analyses in Population Receptive Field Mapping

**DOI:** 10.1002/hbm.70140

**Published:** 2025-01-24

**Authors:** David Linhardt, Michael Woletz, Pedro M. Paz‐Alonso, Christian Windischberger, Garikoitz Lerma‐Usabiaga

**Affiliations:** ^1^ High Field MR Center, Center for Medical Physics and Biomedical Engineering Medical University of Vienna Vienna Austria; ^2^ BCBL ‐ Basque Center on Cognition Brain and Language Donostia ‐ San Sebastián Spain; ^3^ IKERBASQUE Basque Foundation for Science Bilbao Spain

**Keywords:** cortical magnification, fMRI, pRF mapping, retinotopy, visual cortex

## Abstract

Population receptive field (pRF) mapping is a quantitative functional MRI (fMRI) analysis method that links visual field positions with specific locations in the visual cortex. A common preprocessing step in pRF analyses involves projecting volumetric fMRI data onto the cortical surface, typically leading to upsampling of the data. This process may introduce biases in the resulting pRF parameters. Using publicly available analysis containers, we compared pRF maps generated from the original volumetric with those from upsampled surface data. Our results show substantial increases in pRF coverage in the central visual field of upsampled datasets. These effects were consistent across early visual cortex areas V1‐3. Further analysis indicates that this bias is primarily driven by the nonlinear relationship between cortical distance and visual field eccentricity, known as cortical magnification. Our results underscore the importance of understanding and addressing biases introduced by processing steps to ensure accurate interpretation of pRF mapping data, particularly in cross‐study comparisons.

## Graphical abstract

Projecting volumetric fMRI data onto the cortical surface introduces biases in pRF parameters due to upsampling. Using a containerized analysis pipeline, we revealed that upsampling consistently increases pRF coverage in the central visual field, driven by CNR increase and cortical magnification.

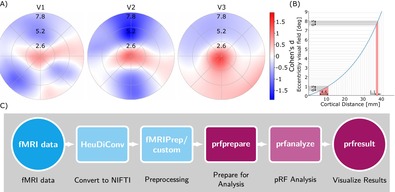

**Summary**

- Spatial upsampling during fMRI data analysis, such as projecting from volume to surface, introduces systematic biases, including increased foveal coverage.
- These biases are driven by enhanced CNR, pRF size increases, and cortical magnification, with distinct contributions from each mechanism.
- The findings emphasize the importance of evaluating processing choices to ensure robust and reproducible pRF mapping results.

## 1 Introduction

The retinotopic organization of the visual system is characterized by the mapping of adjacent positions in the visual field to adjacent positions within the visual cortex (Wandell, Dumoulin, and Brewer 2007). Functional MRI (fMRI) enables us to assess only the collective neural activity within parcellated voxels of the human cortex rather than the responses of individual neurons (Logothetis 2008). However, population receptive field (pRF) mapping enables estimating the combined receptive fields of a population of neurons within a voxel (Dumoulin and Wandell 2008). This approach has been used to assess the organization of the human visual system, showing high reproducibility across repeated acquisitions both in healthy subjects (van Dijk et al. 2016) and in data from subjects stimulated with artificial scotoma (Linhardt et al. 2022). Obtained parameters in pRF mapping can be considered quantitative. However, this assumption is disrupted as every choice made during scanner setup, preprocessing or analysis is influencing the final results.

Traditionally, pRF analysis has been performed in a volumetric space, with each voxel representing a separate time series (Dumoulin and Wandell 2008; Linhardt et al. 2021; Pawloff et al. 2019; Prabhakaran et al. 2021). However, there is a growing trend in pRF mapping studies to project volumetric data onto the cortical surface, where analyses are conducted at the vertex level (Farahbakhsh et al. 2022; Infanti and Schwarzkopf 2020; Morgan and Schwarzkopf 2020; Urale et al. 2022; Himmelberg et al. 2023). Publicly available datasets, such as the NYU dataset (Himmelberg et al. 2021) and the HCP retinotopy data (Benson et al. 2018), also present results projected on a *freesurfer* surface template. This transition to surface analysis offers advantages, such as presenting results on the cortical surface and enabling the unification of vertices across subjects for machine‐learning applications (Ribeiro, Bollmann, and Puckett 2021). However, these benefits come with trade‐offs: surface projection typically involves upsampling, as the number of vertices in general exceeds the original voxel count, and averaging across cortical depths results in the loss of layer‐specific information.

Furthermore, the projection to the surface analysis may introduce potential biases in the pRF results. Interpolating volumetric time‐series data can artificially smooth the data and affect key pRF parameters, such as size, center location, and coverage maps. This study systematically examines these potential biases, emphasizing the need for caution when comparing results obtained using volumetric and surface‐based analyses. We hypothesize that this bias might be due to a combination of three causes: (1) increased contrast‐to‐noise ratio (CNR) in surface compared to volumetric data due to averaging and smoothing; (2) spatial upsampling (more vertices than original voxels) during cortical projection yielding a greater number of pRF centers; and (3) spatial upsampling effects on the results due to the nonlinear mapping from cortex to visual field space (cortical magnification; Daniel and Whitteridge 1961).

To further understand the underlying reasons for this bias, we ran a set of analyses to systematically check our hypotheses: for hypothesis (1), we eliminated the effect of CNR increase by repeating the original analysis on simulated data with varying noise levels. To check for hypothesis (2), we ran two analyses: first, we compared surface and volumetric data with the same number of data points by random sampling of the surface dataset; second, we compared the original surface to a subsampled surface dataset. To test for hypothesis (3), we reproduced the bias by directly upsampling the original 2 mm isotropic voxels to 1 mm isotropic voxels.

## 2 Methods

### 2.1 Participants

A total of 30 right‐handed volunteers (23.8 ± 3.0 years; 16 female) were measured. Participants had normal or corrected‐to‐normal vision and no known ocular pathologies, gave written informed consent, and were financially reimbursed. The study protocol was approved by the ethics committee of the BCBL and complied with the guidelines of the Helsinki Declaration.

### 2.2 Data Acquisition

All scans were performed on an SIEMENS Trio 3 T scanner using a 32‐channel head coil. To minimize movement throughout the scanning sessions, participants' head motions were restricted within the coil using extensive cushioning. Participants were able to see the rear projection screen located outside the scanner bore through a mirror, mounted on top of the head. Functional full‐brain data were acquired using the CMRR multiband EPI sequence (Moeller et al. 2010) with the following parameters: voxel size = 2 × 2 × 2 mm; TR/TE = 1883/30.8 ms; 72 slices; flip angle = 75°; matrix size = 96 × 96; FoV = 192 × 192 mm; phase enc = anterior–posterior; multiband = 3; grappa = 1. Slices of the full‐brain images were aligned parallel with the corpus callosum. Within the scanning session, four runs were acquired per subject, each comprising 135 volumes and lasting 4:14 min. In addition, full‐brain anatomical T1‐weighted scans were acquired with a multiecho (ME) MPRAGE sequence with TE‐s = 1.64, 3.5, 5.36, and 7.22 ms, TR = 2.530 ms, flip angle = 7°, field of view (FoV) = 256 × 256 mm, 176 slices, and voxel size = 1 mm isotropic.

During the functional scans, subjects were presented with a bar aperture (bar width = 1.95°) moving through the visual field in eight different directions revealing an 8 Hz reversing checkerboard pattern. The stimulated field of view was circular, with a radius of 7.8°. Subjects were instructed to maintain central fixation. Fixation was assessed by requiring subjects to report the number of color changes of the central fixation disc overlaid on the bar stimulus. Further information about participants and data acquisition can also be found in (Lerma‐Usabiaga, Carreiras, and Paz‐Alonso 2018).

### 2.3 MRI Preprocessing and Data Analysis

**FIGURE 1:**
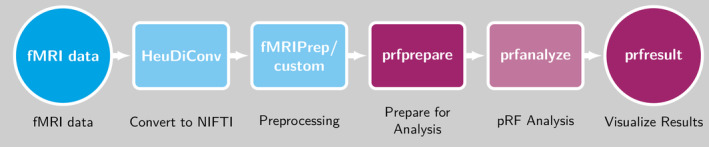
Flowchart of the containerized pipeline for pRF analyses. HeuDiConv converts DICOMs to NIfTI and curates them into BIDS format. fMRIPrep minimally preprocesses the data. prfprepare takes the preprocessed functional data in anatomical or surface space and prepares it for the pRF analysis: it selects only the voxels/vertices in V1‐3 to save computation time. prfanalyze performs pRF analysis using vistasoft. prfresult plots the results as coverage maps or surface overlays.

The fMRI data were analyzed using a series of containerized tools to ensure repeatable results (see Figure 1). An example of subject data processed with the analysis pipeline can be found at https://osf.io/seh6b. It includes all necessary data and scripts as well as the respective analysis results.

As the first step, acquired data were converted to NIfTI format using the containerized version of HeuDiConv v0.11.3 (github.com/nipy/heudiconv). The resulting NIfTi files used in this manuscript were obtained with preprocessing performed using fMRIPrep 23.0.1 (Esteban et al. 2019) and prfprepare. The following description of anatomical and functional data preprocessing has been adapted from the fMRIPrep automatic documentation.

#### 2.3.1 Anatomical Data Preprocessing

T1‐weighted (T1w) images were corrected for intensity nonuniformity (INU) with N4BiasFieldCorrection (Tustison et al. 2010), distributed with ANTs 2.3.3 (Avants et al. 2008). An anatomical reference map was computed after coregistration of two INU‐corrected T1w images (one per session) using mri_robust_template (FreeSurfer 7.3.2, (Reuter, Rosas, and Fischl 2010)). This T1w‐reference image was then skull‐stripped with a NIPYPE 1.8.5 (Gorgolewski et al. 2011) implementation of the antsBrainExtraction.sh workflow (from ANTs), using OASIS30ANTs as the target template. This image was used for brain tissue segmentation into cerebrospinal fluid, white matter, and gray matter using FAST (FSL 6.0.5.1:57b01774, (Zhang, Brady, and Smith 2001)). Brain surfaces were reconstructed using recon‐all (FreeSurfer 7.3.2, (Dale, Fischl, and Sereno 1999)), and the brain masks estimated previously were refined with a custom variation of the method to reconcile ANTs‐derived and FreeSurfer‐derived segmentations of the cortical gray‐matter of Mindboggle (Klein et al. 2017).

#### 2.3.2 Functional Data Preprocessing

For each of the four BOLD runs per subject, the following preprocessing was performed. First, a reference volume and its skull‐stripped version were generated by aligning and averaging one single‐band reference image (SBRef). Head‐motion parameters (transformation matrices) with respect to the BOLD reference were estimated before any spatiotemporal filtering using MCFLIRT(FSL 6.0.5.1:57b01774, (Jenkinson et al. 2002)). BOLD runs were slice‐time corrected to 0.906 s (center of slice acquisition range 0–1.81 s) using the 3dTshift routine included in AFNI (Cox and Hyde 1997). BOLD time series were resampled by applying the transforms to correct for head motion. These resampled BOLD time‐series will be referred to as preprocessed BOLD in original space, or just preprocessed BOLD.

BOLD reference data were then co‐registered to the T1w reference image using bbregister (FreeSurfer) which implements boundary‐based registration (Greve and Fischl 2009). Co‐registration was configured with six degrees of freedom. First, a reference volume and its skull‐stripped version were generated using a custom methodology of fMRIPrep. The BOLD time series were resampled onto the fsnative and fsaverage surfaces, where fsnative is the individual subject surface representation and fsaverage is based on the MNI305 surface representation. All resamplings were performed with a single interpolation step by composing all the pertinent transformations (i.e., head‐motion transform matrices, susceptibility distortion correction when available, and co‐registrations to anatomical and output spaces). Gridded (volumetric) resamplings were performed using antsApplyTransforms (ANTs), configured with Lanczos interpolation to minimize the smoothing effects of other kernels (Lanczos 1964). Nongridded (surface) resamplings were performed using mri_vol2surf (FreeSurfer). The call of mri_vol2surf in fMRIPrep uses multiple surfaces in distances of 0.2, between 0 (white—gray matter surface) and 1 (grey matter—pial surface). For each vertex, the values along the surface normal are calculated using trilinear interpolation and averaged across cortical depth. Many internal operations of fMRIPrep use Nilearn 0.9.1 (Abraham et al. 2014), mostly within the functional processing workflow.

#### 2.3.3 Functional Data Analysis

Following minimal preprocessing, functional data were further processed using the prfprepare container v1.3.5 (github.com/fmriat/prfprepare). This container prepares both stimuli and functional data for input into subsequent pRF analysis. Specifically, it generates stimuli in NIfTI format from vistadisp stimulus presentation suite log files (github.com/vistalab/vistadisp) and masks functional data based on cortical atlases, either Wang (Wang et al. 2015) or Benson (Benson et al. 2014). For this masking, the Neuropythy tool (Benson and Winawer 2018; github.com/noahbenson/neuropythy) was used. For all subsequent analyses, the Benson atlas definitions of visual areas were employed. This step preparing the data for the analysis was performed, in both volume space (original voxel) and surface space (Freesurfer's fsnative space) as provided by fMRIPrep. The masked data were then saved as a single NIfTI file per subject, run, and hemisphere, containing all time courses of the masked voxels/vertices for pRF analysis and averaged across all runs. Additionally, a sidecar JSON file was saved for each defined region of interest (ROI), specifying the indices for the corresponding ROI and their positions in the original volumetric or surface file. This information was later used to reconstruct the original file structure and group data by ROI.

Subsequently, pRF analysis was conducted using the containerized solution prfanalyze, originally presented in Lerma‐Usabiaga et al. (2020) and publicly available (github.com/vistalab/prfmodel). While this set of containers offers multiple analysis tools with consistent input and output formats, the vistasoft (github.com/vistalab/vistasoft) implementation was chosen for the current study (prfanalyze‐vista v2.2.1_3.1.0). Fitting is performed using the single‐Gaussian model in a coarse‐to‐fine approach. The prfanalyze container was slightly modified to perform the analysis on subject‐specific data.

The resulting pRF parameters were accessed and analyzed using a Python interface for the standardized output of prfanalyze, available at github.com/dlinhardt/prfclass. Based on this code, the prfresult container v0.0.6 was used to reconstruct the original volume and surface files and visualize pRF parameters as coverage plots and cortical overlays.

### 2.4 Simulations and Statistical Analyses

#### 2.4.1 Coverage Map Analysis

**FIGURE 2:**
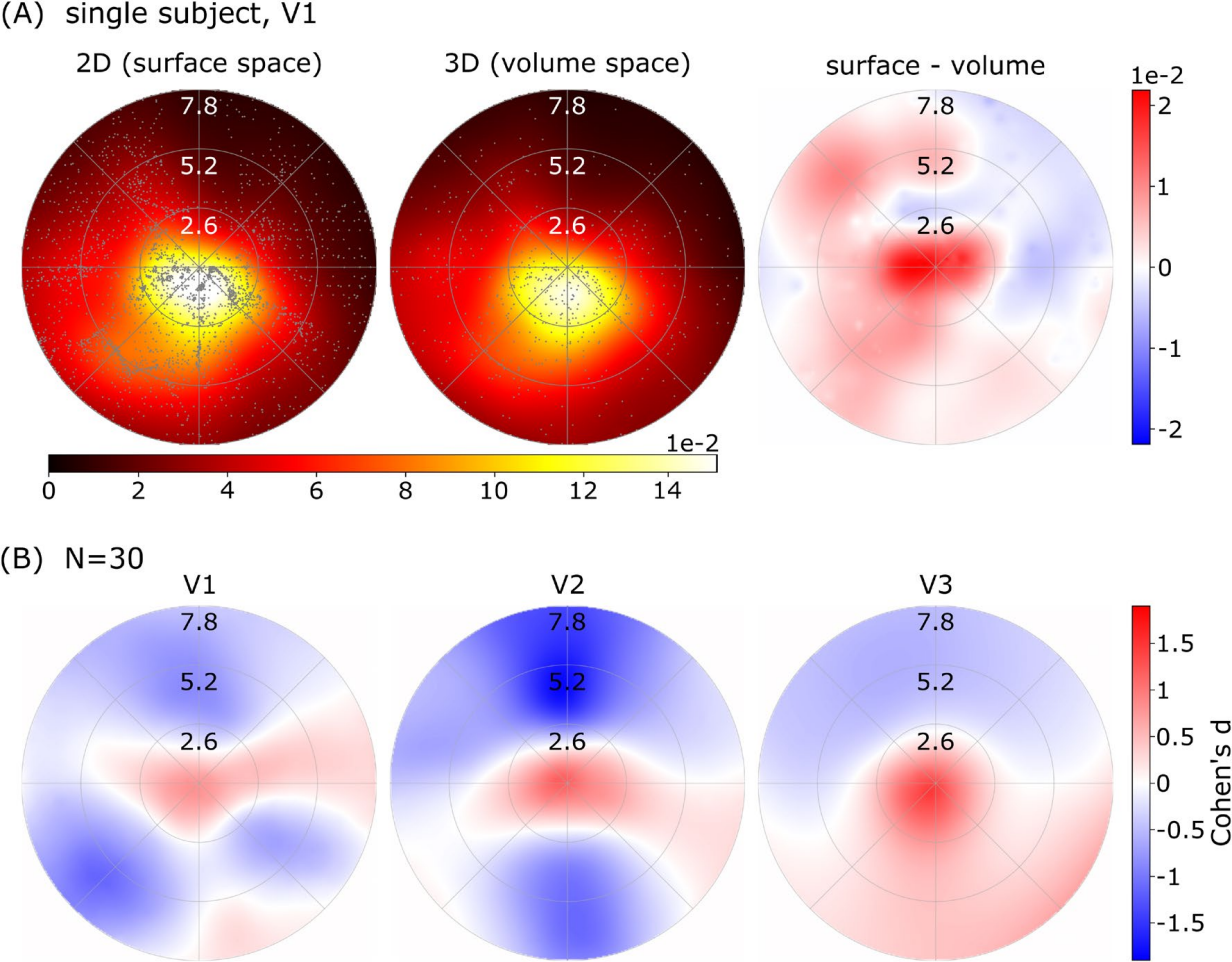
Coverage plots based on above‐threshold voxels/vertices. A. Coverage plots for surface (left) and volumetric (right) data for V1 in a representative subject. There are substantially more pRF centers (gray dots) in the surface results. The difference between the two coverages can be seen on the right. Already in this single subject, we can see a central coverage bias. B. For the group‐level comparison (*N* = 30), the coverage differences between surface and volume analyses show a higher foveal representation in the surface data. Effect sizes (Cohen's d) are shown for V1, V2, and V3, where red indicates greater coverage in the surface data. The observed systematic effect is only clearly visible across the whole group of subjects.

As the direct mapping between the volumetric and surface analysis spaces cannot be established easily, we employed a comparison of the resulting visual field coverage maps. Visual field coverage maps (Amano, Wandell, and Dumoulin 2009) were created independently for every run and condition (volume and surface results) as follows. Every voxel/vertex is represented by a two‐dimensional Gaussian function in visual field space. This Gaussian is defined using the fitted parameters for center position (x, y) and pRF size (σ) with a maximum height of one. The coverage map is calculated as the average value at every visual field location, taking into account all above threshold voxels. In Figure 2, coverage is represented by the color‐coded value in the background, while gray dots show every pRF center fitted. If not stated otherwise, the data were thresholded using a minimum variance explained of 20%. Disparities between these coverage maps were calculated by evaluating paired Cohen's d at each position in the coverage plot. For a robust group‐level analysis, we employed a bootstrapping method across single‐subject Cohen's d values, executing 50 iterations with replacement. The analysis scripts for these analyses are available at github.com/garikoitz/prf2d3d.

#### 2.4.2 Simulations

In addition to the experimental pRF data, we also wanted to examine the effects of CNR and pRF sizes on the resulting maps. For this, we created 30 artificial subject datasets, with the same number of data points as acquired in the experimental data and simulated results for volumetric and surface processing. pRF center positions were drawn from a Gaussian distribution in the visual field, centered at the fovea. Single‐subject coverage plots were calculated for both conditions and Cohen's d effects were calculated, mimicking the analyses in the experimental data. To isolate the effect of CNR differences, we kept the pRF size constant while reducing CNR in the volumetric data in multiple steps. To isolate the effect of different pRF sizes, we kept CNR constant in both datasets, while increasing pRF size in the surface set in three steps.

#### 2.4.3 Noiseless Data

For the creation of noiseless datasets, we used previously calculated pRF parameters from the original analysis to compute the fitted model time series. We then substituted each voxel's time series in the original volumetric data with these modeled noise‐free time series. The procedure is similar to creating noiseless synthetic data where the spatial distribution of the ground truth parameters is known. Similar to the procedure used in the initial analyses, data were projected onto the surface using Freesurfer's *mri_vol2surf* function. Parameters were matched to those in the standard fMRIPrep implementation to maintain consistency across analyses. Results for the volumetric analyses should perfectly recover the same parameters used for the model creation (Lerma‐Usabiaga et al. 2020). The substitution was performed to create 30 simulated subject datasets. As these datasets are noiseless, we expect close to perfect fits, so we used a threshold of 50% variance explained.

#### 2.4.4 Equating the Number of Voxels

To assess whether the observed bias is driven by the differing number of above‐threshold data points between volumetric and surface analyses, we performed a comparison using an equalized number of data points. For this, we used the noiseless dataset described above. Specifically, we randomly subsampled the surface data to match the number of vertices to the number of voxels in the volumetric analysis for each subject. In the first analysis, the subsampled surface data were compared directly to the original volumetric data, both of which contained the same number of pRFs. In the second analysis, we compared the original surface data with the subsampled surface data, mimicking the difference in voxels and vertices observed in the initial volume‐to‐surface comparison.

#### 2.4.5 Spatial Upsampling

For testing whether the upsampling step is driving the bias, the original volumetric data were spatially upsampled by reslicing from 2 to 1 mm isotropic resolution. A trilinear interpolation kernel was used as implemented in the AFNIs *3dresample* tool (Cox 1996).

#### 2.4.6 Cortical Location Function

The nonlinear relationship between a position in the visual cortex and the eccentricity in the visual field is modeled as an exponential function according to (Schwartz 1977, 1980; Strasburger 2022) and called the cortical location function (CLF) (Equation 1)

(1) $$E\left(d\right)=E_{2}\;\left(\exp\left(\frac{d}{M_{0}E_{2}}\right)-1\right)$$

where E is the eccentricity in the visual field, and d is the distance from the occipital pole on the cortex. The constant *M*_0_ is called the cortical magnification factor and $E_{2}$ the eccentricity value at which the inverse $M^{-1}$ doubles the foveal value.

To estimate the empirical CLF for our dataset, we first calculated the cortical magnification function (CMF), based on the moving ring method (as described in github.com/noahbenson/cortical‐magnification‐tutorial). Surface data were thresholded to include only vertices with at least 10% variance explained and a maximum eccentricity of 9°. The square root of the resulting empirical distribution was taken, and the linear CMF was then fitted according to Equation (2), with the cortical scaling factor A (Dougherty et al. 2003; Harvey and Dumoulin 2011; Schira et al. 2010).

(2) $$M\left(E\right)=\frac{A}{E+E_{2}}$$

We used this fitted function to calculate the cortical magnification factor $M_{0}=M\left(0\right)$ in our experimental dataset.

## 3 Results

The functional data were acquired at a 2 mm isotropic resolution. Converting this data to the cortical surface yielded approximately 4.4 times more vertices on the surface of V1 than there were voxels in the volume. This behavior is expected because the resolution of the cortical surface to which the data are projected is predefined. This ratio increased further after fitting pRF parameters and applying a goodness‐of‐fit threshold (variance explained; VE). Once thresholded independently, there were approximately 5.8 times more surviving vertices in surface V1 compared to voxels in the volumetric V1, indicating that relatively more vertices survive the VE threshold in the surface data. Relatively, 56% of vertices on the surface are masked, compared to 67% of voxels in the volume, when applying the same variance explained threshold of 20%. To achieve an equivalent percentage of masked vertices on the surface, the variance explained threshold would need to be increased to 41%.

Figure 2 illustrates the initial bias we found when comparing pRF mapping results obtained from analyses in volumetric and surface space, both thresholded for 20% variance explained. In Figure 2A, we show the V1 coverage maps for both surface and volumetric results in one representative subject as well as the coverage differences. It can be seen that the number of pRF centers (gray dots) present in the surface results is greatly increased compared to the volumetric variant. The difference between the two coverage plots (right) already shows a foveal coverage bias for the surface result. In Figure 2B, we compare surface‐volumetric coverage for visual areas V1, V2, and V3 at the group level (*N* = 30). The bootstrapped effect size of the difference (Cohen's d) is shown for every location of the visual field (represented as a mesh of 128 × 128 pixels). In particular, the foveal part of the visual field yielded higher coverage for the results corresponding to the surface representation. More peripheral areas of the visual field show the opposite effect. Effect sizes of the foveal bias increase in higher visual areas (V2, V3). Note that the results in Figure 2 use the same variance explained threshold of 20% for both volume and surface, as is typically done in retinotopic experiments. When the same percentage of voxels and vertices is masked (using 20% for volume and 41% for surface), the coverage bias even increases (see Figure S1B).

We derived three hypotheses potentially explaining the observed effect: (1) increased CNR in surface compared to volumetric data due to averaging and smoothing; (2) spatial upsampling during cortical projection yielding a greater number of pRF centers; (3) spatial upsampling effects due to the nonlinear CLF, which maps positions in the visual cortex to locations in the visual field.

### 3.1 Effect of CNR Differences

**FIGURE 3:**
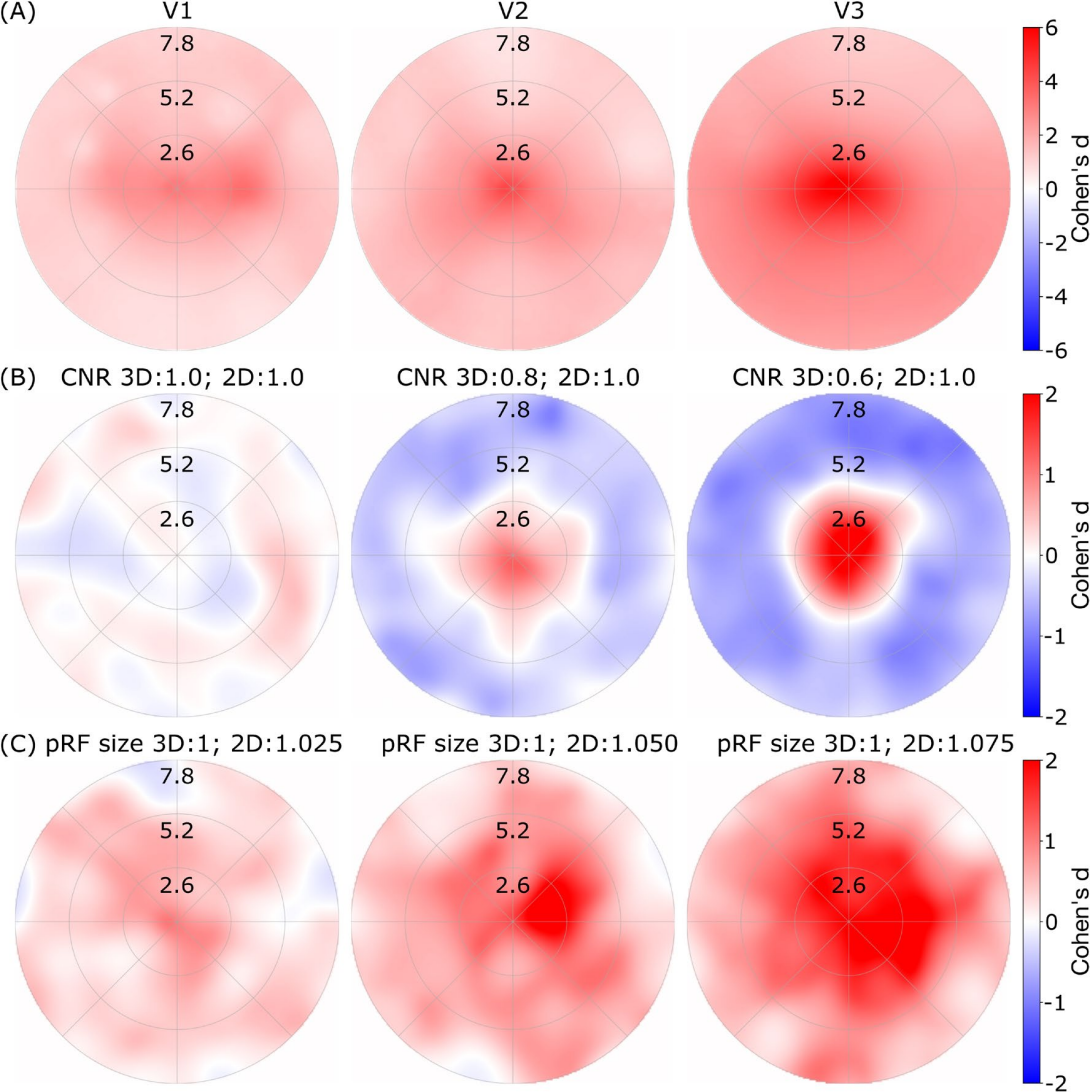
Group coverage surface‐volumetric differences in noiseless data and simulations to check for CNR and pRF size effects. Bootstrapped (50 repetitions with replacement) effect size (Cohen's d) representation of the surface‐volume difference in field of view coverage depending on the different analyses. The comparisons were paired. A. Noiseless data in volumetric versus its projection to the surface. Only voxels with more than 50% variance explained were included. B. Simulations to examine CNR effects. pRF size between the two conditions was kept constant, while the CNR differences were increased from left to right. With no CNR difference, the Cohen's d map equals a random field (leftmost panel). With CNR differences, a clear bias toward higher coverage in the fovea and lower coverage in the periphery appears (center and right panels). C. Simulations to examine pRF size effects. CNR was equated in the two conditions, but the pRF size for the surface condition was increased from left to right. With increasing difference a similar bias occurs, however, the bias in the foveal compared to peripheral areas was less pronounced.

The first hypothesis (1) proposes that differences in CNR are the driving factor behind the observed bias. CNR measures the ability to distinguish signal from noise in the data and is increased when volumetric data are projected onto the cortical surface, as random noise is averaged out during interpolation. To test this hypothesis, we eliminated the effect of CNR differences by using synthesized noiseless time‐series data. The results of this analysis, shown in Figure 3A, show that coverage of surface analysis results is higher in the whole visual field throughout all early visual areas, as indicated by the absence of negative Cohen's d values. Nonetheless, the pattern of increased coverage in the central visual field, seen in the experimental data, remains clearly visible. This suggests that increased CNR is not the primary driver of the observed foveal bias.

While interpolation increases CNR, it also comes at the cost of signal blurring. In pRF mapping, this is particularly significant because neighboring voxels, due to retinotopic organization, do not have perfectly similar time courses. Their time‐course peaks are slightly shifted, as each voxel corresponds to a different part of the visual field. Averaging during interpolation alters the resulting time course and can influence the estimated pRF parameters. To isolate the effects of CNR increases and pRF size changes, we conducted two simulations. In the first (Figure 3B), we kept pRF size constant while varying CNR. We found that similar CNR values (left) show no systematic bias. Increasing CNR differences (center and right) resulted in a central coverage bias similar to the experimental data. In contrast, increasing pRF size caused a broader increase in coverage across the visual field, suggesting that pRF size changes may contribute as well but do not fully explain the observed bias (Figure 3C).

As an additional analysis, we transformed the pRF parameters obtained in volume space to the cortical surface using Freesurfer's *vol2surf*. This was done using options similar to the original processing. Figure S1C compares the results obtained in the volume with the projected pRF parameters. The overall coverage difference is reduced compared to Figure 3A, though a slight foveal bias remains.

To further investigate how interpolation methods influence the observed bias, we repeated the analysis using nearest neighbor interpolation instead of trilinear interpolation during the surface projection of the noiseless data. The results, presented in Figure S1A, show a reduction in bias compared to trilinear interpolation, which we attribute to the absence of pRF size increases typically introduced through averaging with the trilinear method.

### 3.2 Equating the Number of Voxels

**FIGURE 4:**
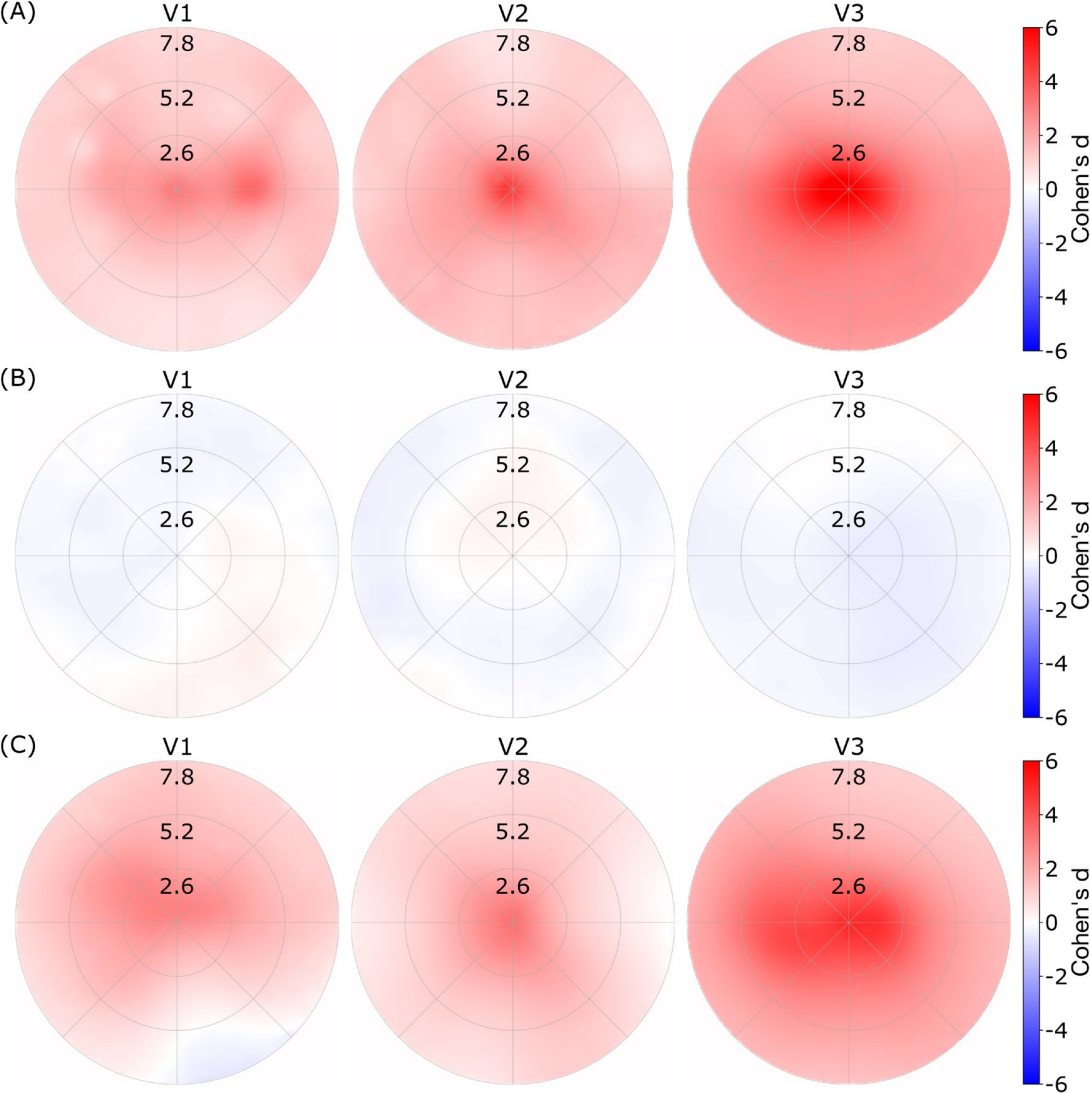
Upsampling effects on the differences between surface and volumetric processing. A. Subsampled surface results compared to the full volumetric results, with an equal number of voxels/vertices. This analysis reproduces the central visual field bias. Thus, the number of data points is not the reason for the bias seen in the experimental results. B. Comparison of the surface data with a random subsample of the same data. No difference can be found in this analysis. C. Upsampled volumetric images (reslicing the voxels from 2 mm isotropic to 1 mm isotropic) compared to the original volumetric dataset. Here the same bias occurs as in panel A. To summarize, regardless of the number of data points, if there is upsampling (volumetric to surface or volumetric to volumetric), there is a foveal bias effect.

In hypothesis (2), we tested whether the large difference in the number of above‐threshold voxels and vertices drives the central coverage bias by conducting two separate analyses (Figure 4). The first analysis compared the subsampled surface data to the volumetric data, both containing an equal number of pRFs. The results (Figure 4A) revealed a central coverage bias nearly identical to that observed in the original noiseless results (Figure 3A). In the second analysis, we compared the original surface data to the subsampled surface data (Figure 4B), showing almost no difference between the two.

These findings indicate that the observed bias stems from the spatial distribution of pRF centers rather than the absolute number of pRFs. Specifically, the spatial upsampling during projection to the cortical surface appears to be the primary factor driving the bias.

### 3.3 Spatial Upsampling

Hypothesis (3) states that the observed effect is due to the upsampling of data itself. For testing this, results for the upsampled volumetric data were compared to the low‐resolution original volumetric data (Figure 4C). Again, a pronounced effect is observed, with the spatially upsampled data having greater coverage in the central visual field compared to the original, not resliced data.

This bias is likely caused by the nonlinearities in the relationship between cortical location and eccentricity in the visual field. This nonlinearity is characterized by the cortical magnification (Daniel and Whitteridge 1961) of the visual system.

**FIGURE 5:**
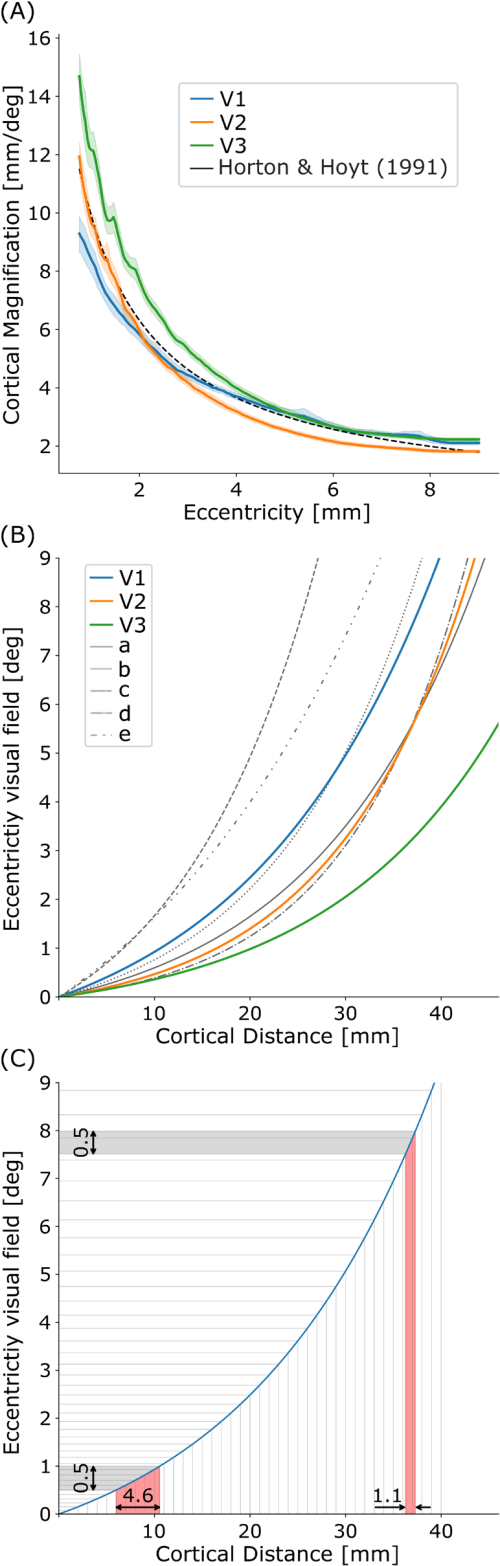
A. Linear cortical magnification function (CMF) for the early visual cortex areas V1 (blue), V2 (orange), and V3 (green) with their 95% confidence intervals, overlaid with previously published results (black) from (Horton and Hoyt 1991). The functions represent the square root of the estimated empirical areal CMF. B. CLF on our experimental data pRF results for V1 (blue), V2 (orange), and V3 (green). For comparison, the gray lines represent V1 results as reported by Strasburger (2022); Strasburger, Rentschler, and Juttner (2011) and based on different studies (a: (Larsson and Heeger 2006), b: (Duncan and Boynton 2003), c: (Cowey and Rolls 1974), d: (Schira et al. 2009), e: (Dougherty et al. 2003)). C. CLF of the primary visual cortex V1 with an exemplary regular sampling of the cortical distance (same distance on *x*‐axis). The colored bands indicate that the same amount of visual angle (gray) corresponds to different areas of the cortex (red). The sampling in the foveal areas is considerably denser than in more peripheral parts. When the data on the cortical level are linearly upsampled, the density difference between foveal and peripheral areas is intensified, leading to the effect observed in this study.

The obtained values for $E_{2}$ [deg] are for V1: 1.4; V2: 0.5; V3: 0.5. The obtained $M_{0}$ values [mm/deg] for the early visual cortex are for V1: 14.0; V2: 30.8; V3: 36.9. Plots of the cortical magnification factors over eccentricity are shown in Figure 5A with reference data from the literature (Horton and Hoyt 1991). Figure 5B shows the CLF (Equation 1) for the obtained values for V1 (blue continuous line), V2 (orange continuous line), and V3 (green continuous line). For comparison, we plotted values taken from the literature in gray (Strasburger, Rentschler, and Juttner 2011; Strasburger 2022). It can be seen that our experimentally obtained model for V1 is compatible with previously published results.

In Figure 5C, we illustrate how the cortical location function (CLF) maps positions on the visual cortex to locations in the visual field, emphasizing its exponential nature. When data are spatially upsampled, for the same amount of new positions on the cortex (*x*‐axis), the relative contribution of these new points in the visual field is overrepresented in the fovea (*y*‐axis). The opposite happens in the periphery.

Our results indicate that the foveal bias arises from a combination of three effects originating from the data upsampling process. While the absolute number of voxels does not influence the bias (Figure 4A,B), our analyses and simulations (Figure 3) demonstrate that averaging introduces biases through increased CNR and changes in pRF sizes. Additionally, we found that independent of the CNR bias, the exponential mapping function between visual cortex positions and visual field locations (CLF) is the primary driver of the increased coverage in foveal regions and reduced coverage in peripheral areas.

## 4 Discussion

In this work, we studied disparities in pRF‐based visual field coverage maps when applying spatial transformation procedures: volume‐to‐surface and volume‐to‐volume upsampling. After the volume‐to‐surface transformation, we found an increase in pRF coverage in the fovea compared to the periphery (Figure 2). We characterized and isolated these effects in several analyses, and concluded that differences were due to a combination of two factors: increase in CNR (hypothesis 1) and cortical magnification (hypothesis 3). The number of data points, however, did not have an effect (hypothesis 2).

Upsampling data (both volume‐to‐surface and volume‐to‐volume) lead to increases in CNR due to averaging data from neighboring voxels. The averaging process smooths the time courses, also leading to an increase in pRF sizes fitted. The increase in CNR and pRF sizes partially explains the effect of increased foveal coverage observed in this study, through different mechanisms. The increase in CNR enhances the likelihood of vertices on the surface exceeding the variance‐explained threshold. Consequently, a smaller proportion of voxels in the volume space surpass this threshold.

The first mechanism is related to the increase in CNR and its influence on the distribution of pRF centers in the visual field. The distribution of pRF centers is not uniform across the visual field, as there is a higher density in the foveal regions. When using fMRI and pRF mapping algorithms, noise introduces a broader probability distribution for each pRF center. Instead of being Gaussian, these probability distributions are skewed, pulling the centers toward more eccentric (peripheral) regions. As a result, the overall distribution of pRF centers across the visual field becomes wider, with more centers being inaccurately positioned toward the periphery. Due to the smoothing in the spatial upsampling operation, noise is reduced and the pRF center distribution is changed, again narrowing the distribution with a stronger peak and lower values in the periphery. When we now compare the unsmoothed with the smoothed distributions, we expect the results shown in Figure 2: more coverage in the center and less coverage in the periphery. Through simulations (Figure 3B) we isolated this partial effect.

A second mechanism is related to the fact that both types of upsampling are increasing the pRF size and therefore affecting the coverage. Temporal filtering using a low‐pass filter, equivalent to smoothing of the time course, leads to peak widening and pRF size increases (Morgan and Schwarzkopf 2020). This process systematically affects the whole visual field but is more prominent in foveal regions. The effect of size increase was isolated in a separate simulation, maintaining constant CNR while varying pRF size (Figure 3C). This finding confirmed that pRF size plays a role in explaining the observed main effect, although its contribution is much smaller than the CNR increase.

In hypothesis (2), we considered the possibility that the difference in coverage is attributed to the substantial difference in voxel/vertex number between the upsampled and nonupsampled datasets. A priori, this should not be the case, given the way coverage maps are calculated, as values across all pRFs are averaged in every pixel of the visual field. To test this, we conducted two analyses. First, we randomly subsampled the surface data to match the size of the original volumetric data, showing results consistent with the noiseless comparison (see Figure 4A). Second, we compared the original surface data with the subset of the same surface data, and observed no discernible differences (Figure 4B). These findings indicate that variations in coverage are not due to the number of data points but are instead influenced by differences in the underlying sample distributions. This is further highlighted in Figure S1B, where applying a higher threshold to the surface data is even slightly increasing the observed central coverage bias, confirming the presence of differing distributions.

Our volume‐to‐volume upsampling analysis, and particularly the effects of cortical magnification (Daniel and Whitteridge 1961), supports our hypothesis (3) that both spatial upsampling and cortical magnification contribute to the foveal coverage bias. However, the volume‐to‐volume upsampling analysis essentially replicates the original volume‐to‐surface effect without isolating the possible contribution of cortical magnification. To understand the underlying mechanism, we used the same experimental data to estimate the CLF (see Figure 5B), which links distinct positions on the cortex to positions on the visual field. We hypothesized that the experimental data would reflect this function. Our data showed a comparable CMF ($M_{0}$) close to previously reported values, with increasing cortical magnification factors in higher visual areas. This increase in CMF explains our results showing a bigger effect for higher visual areas V2 and V3 than for V1 (see Figures 2, 3, 4). Previously reported CMF were not consistent when comparing visual areas, as they showed a reduction (Dougherty et al. 2003; Horton and Hoyt 1991), an increase (Silva et al. 2018), and a difference that changes depending on the eccentricity (Benson et al. 2022; Himmelberg et al. 2023; Schira et al. 2009, 2010). CMFs obtained in the current study are consistent with the recent literature.

Thus, the third contributing factor to the increased foveal coverage after both spatial upsampling methods, whether directly in the space of the volume or by projecting it onto the surface, is the exponential relationship between visual field eccentricity and cortical location (Keliris et al. 2019). This CLF and its effect is visualized in Figure 5C. When positions on the cortex (*x*‐axis) are linearly upsampled, the effect is exponentially distributed in the visual field (*y*‐axis). This leads to a nonlinear increase in pRF center density in the central visual field relative to peripheral areas. Spatial upsampling of the data results in a more fine‐grained sampling of the cortex and is equivalent to halving each interval on the *x*‐axis of Figure 5C (gray lines). Though each subsampled step will have a similar distance to its neighbors on the *x*‐axis, the exponential transformation will bring the corresponding position in the visual field slightly closer to the foveal neighbor than to the peripheral. Consequently, this causes an overall shift of the pRF center distribution toward the central visual field. Additionally, the exponential function increases the sampling density for the central visual field, while lowering it in the periphery. This effect describes the underlying distribution of pRF centers in the visual field, peaking in the center.

In contrast to qualitative analyses such as GLM, in pRF mapping, we typically consider the resulting parameters as quantitative. However, as our findings demonstrate, even minor changes in analysis choices can disrupt this assumption. Every choice made during data processing will influence final results, and researchers should be aware of and consider tradeoffs associated with every step and how it may alter the final analysis. Recognizing this is critical, especially when aiming for cross‐study comparisons or clinical applications. In studies on scotoma patients, this bias could distort measurements of scotoma size and shape, leading to potential misclassification or misinterpretation of visual deficits when using pRF data. Though this bias is less problematic for within‐study comparisons where preprocessing steps remain consistent, it complicates cross‐study comparisons.

In modern fMRI analyses, there are several reasons for upsampling the data. In studies involving multimodal data, reslicing is used to unify resolution across modalities, that is, bringing diffusion MRI (dMRI), quantitative MRI (qMRI), and fMRI into the common T1w isotropic space (Lerma‐Usabiaga, Carreiras, and Paz‐Alonso 2018). Often, the other modalities are resliced to the high‐resolution anatomical T1w template. Another common application of upsampling is the initially discussed situation where the data are upsampled when projecting volumetric time series to the surface mesh. Working on the surface has previously shown advantages (freesurfer.net; Fischl 2012), and many current fMRI analysis pipelines include this step. In standard fMRI retinotopy analyses, the number of vertices in the surface approach is usually substantially higher than the original number of acquired voxels. Researchers must carefully interpret upsampled results, taking into account the systematic biases revealed in this study, as well as other potential biases introduced by data processing steps such as interpolation, smoothing, and transformation of voxel data to surface space.

A limitation of this study is that the observed effect of increased foveal coverage was not demonstrated in data acquired with different voxel sizes. Future studies should address this question, although we consider this comparison complex. Together with changes in voxel size, many other sequence parameters will change, making it challenging to isolate the voxel size effect alone.

In pRF mapping, the parameters are considered quantitative. Biases introduced by differing experimental or analytical pipelines may not be problematic within individual studies or labs but pose challenges when comparing results across labs. Our findings underscore that even minor analysis choices can disrupt the assumption that pRF parameters are quantitative, complicating cross‐study or cross‐site comparisons. The introduced systematic differences can easily be misinterpreted as genuine experimental effects. This concern is particularly relevant when comparing data to reference datasets like the HCP retinotopy dataset (Benson et al. 2018). Recognizing and addressing these biases is critical to ensure robust and reliable pRF analyses.

## 5 Conclusion

In conclusion, we have demonstrated that different processing strategies can have a considerable impact on the results of pRF mapping analyses. As a quantitative fMRI technique, pRF mapping offers some advantages over classical GLM analyses. However, as our understanding of the technique evolves, we become aware of how different aspects of the analyses may influence the results. Our study revealed systematic biases introduced when transitioning from volumetric to surface analytical spaces in pRF mapping, or when spatially upsampling. These findings have significant implications for the analytical decisions and interpretation of results in neuroimaging studies and warrant further investigation. The tools presented in this work will be valuable for future researchers investigating methodological or scientific questions in the field of pRF mapping.

## Supporting information


**Figure S1.** Cohen’s d comparisons for V1‐3 and different analyses: A. Noiseless data analyzed projected to the surface using a nearest‐neighbor approach compared to volume analysis. The overall bias is slightly reduced compared to Figure 3A but remains present. B. Surface data thresholded at 41% variance explained compared to volume data with 20% threshold. The foveal bias is slightly increased compared to Figure 2B. C. Noiseless data were analyzed in the volume space with pRF parameters subsequently projected to the surface. This approach reduces the bias compared to projecting time courses to the surface and analyzing them there, as in Figure 3A.

## Data Availability

The data that support the findings of this study are available from the corresponding author upon reasonable request.

## References

[hbm70140-bib-0001] Abraham, A. , F. Pedregosa , M. Eickenberg , et al. 2014. “Machine Learning for Neuroimaging With Scikit‐Learn.” Frontiers in Neuroinformatics 8: 00014. https://www.frontiersin.org/articles/10.3389/fninf.2014.00014. 10.3389/fninf.2014.00014 PMC3930868 24600388

[hbm70140-bib-0002] Amano, K. , B. A. Wandell , and S. O. Dumoulin . 2009. “Visual Field Maps, Population Receptive Field Sizes, and Visual Field Coverage in the Human MT+ Complex.” Journal of Neurophysiology 102, no. 5: 2704–2718. 10.1152/jn.00102.2009. 19587323 PMC2777836

[hbm70140-bib-0003] Avants, B. , C. L. Epstein , M. Grossman , and J. C. Gee . 2008. “Symmetric Diffeomorphic Image Registration With Cross‐Correlation: Evaluating Automated Labeling of Elderly and Neurodegenerative Brain.” Medical Image Analysis 12, no. 1: 26–41. 10.1016/j.media.2007.06.004. 17659998 PMC2276735

[hbm70140-bib-0054] Benson, N. C. , and J. Winawer . 2018. “Bayesian Analysis of Retinotopic Maps.” eLife 7: e40224. 10.1101/325597. 30520736 PMC6340702

[hbm70140-bib-0005] Benson, N. C. , J. M. D. Yoon , D. Forenzo , S. A. Engel , K. N. Kay , and J. Winawer . 2022. “Variability of the Surface Area of the V1, V2, and V3 Maps in a Large Sample of Human Observers.” Journal of Neuroscience 42, no. 46: 8629–8646. 10.1523/JNEUROSCI.0690-21.2022. 36180226 PMC9671582

[hbm70140-bib-0004] Benson, N. C. , K. W. Jamison , M. J. Arcaro , et al. 2018. “The Human Connectome Project 7 Tesla Retinotopy Dataset: Description and Population Receptive Field Analysis.” Journal of Vision 18, no. 13: 23. 10.1167/18.13.23. PMC6314247 30593068

[hbm70140-bib-0053] Benson, N. C. , O. H. Butt , D. H. Brainard , and G. K. Aguirre . 2014. “Correction of Distortion in Flattened Representations of the Cortical Surface Allows Prediction of V1‐V3 Functional Organization from Anatomy.” PLoS Computational Biology 10, no. 3: e1003538. 10.1371/journal.pcbi.1003538. 24676149 PMC3967932

[hbm70140-bib-0006] Cowey, A. , and E. T. Rolls . 1974. “Human Cortical Magnification Factor and Its Relation to Visual Acuity.” Experimental Brain Research 21, no. 5: 447–454. 10.1007/BF00237163. 4442497

[hbm70140-bib-0007] Cox, R. W. 1996. “AFNI: Software for Analysis and Visualization of Functional Magnetic Resonance Neuroimages.” Computers and Biomedical Research 29, no. 3: 162–173. 10.1006/cbmr.1996.0014. 8812068

[hbm70140-bib-0008] Cox, R. W. , and J. S. Hyde . 1997. “Software Tools for Analysis and Visualization of fMRI Data.” NMR in Biomedicine 10, no. 4–5: 171–178. 10.1002/(sici)1099-1492(199706/08)10:4/5<171::aid-nbm453>3.0.co;2-l. 9430344

[hbm70140-bib-0009] Dale, A. M. , B. Fischl , and M. I. Sereno . 1999. “Cortical Surface‐Based Analysis.” NeuroImage 9, no. 2: 179–194. 10.1006/nimg.1998.0395. 9931268

[hbm70140-bib-0010] Daniel, P. M. , and D. Whitteridge . 1961. “The Representation of the Visual Field on the Cerebral Cortex in Monkeys.” Journal of Physiology 159, no. 2: 203–221. 10.1113/jphysiol.1961.sp006803. 13883391 PMC1359500

[hbm70140-bib-0011] Dougherty, R. F. , V. M. Koch , A. A. Brewer , B. Fischer , J. Modersitzki , and B. A. Wandell . 2003. “Visual Field Representations and Locations of Visual Areas V1/2/3 in Human Visual Cortex.” Journal of Vision 3, no. 10: 1. 10.1167/3.10.1. 14640882

[hbm70140-bib-0012] Dumoulin, S. O. , and B. A. Wandell . 2008. “Population Receptive Field Estimates in Human Visual Cortex.” NeuroImage 39, no. 2: 647–660. 10.1016/j.neuroimage.2007.09.034. 17977024 PMC3073038

[hbm70140-bib-0013] Duncan, R. O. , and G. M. Boynton . 2003. “Cortical Magnification Within Human Primary Visual Cortex Correlates With Acuity Thresholds.” Neuron 38, no. 4: 659–671. 10.1016/S0896-6273(03)00265-4. 12765616

[hbm70140-bib-0014] Esteban, O. , C. J. Markiewicz , R. W. Blair , et al. 2019. “fMRIPrep: A Robust Preprocessing Pipeline for Functional MRI.” Nature Methods 16, no. 1: 111–116. 10.1038/s41592-018-0235-4. 30532080 PMC6319393

[hbm70140-bib-0015] Farahbakhsh, M. , E. J. Anderson , R. O. Maimon‐Mor , et al. 2022. “A Demonstration of Cone Function Plasticity After Gene Therapy in Achromatopsia.” Brain 145, no. 11: 3803–3815. 10.1093/brain/awac226. 35998912 PMC9679164

[hbm70140-bib-0016] Fischl, B. 2012. “FreeSurfer.” NeuroImage 62, no. 2: 774–781. 10.1016/j.neuroimage.2012.01.021. 22248573 PMC3685476

[hbm70140-bib-0017] Gorgolewski, K. , C. D. Burns , C. Madison , et al. 2011. “Nipype: A Flexible, Lightweight and Extensible Neuroimaging Data Processing Framework in Python.” Frontiers in Neuroinformatics 5: 00013. https://www.frontiersin.org/articles/10.3389/fninf.2011.00013. 10.3389/fninf.2011.00013 PMC3159964 21897815

[hbm70140-bib-0018] Greve, D. N. , and B. Fischl . 2009. “Accurate and Robust Brain Image Alignment Using Boundary‐Based Registration.” NeuroImage 48, no. 1: 63–72. 10.1016/j.neuroimage.2009.06.060. 19573611 PMC2733527

[hbm70140-bib-0019] Harvey, B. M. , and S. O. Dumoulin . 2011. “The Relationship Between Cortical Magnification Factor and Population Receptive Field Size in Human Visual Cortex: Constancies in Cortical Architecture.” Journal of Neuroscience 31, no. 38: 13604–13612. 10.1523/JNEUROSCI.2572-11.2011. 21940451 PMC6623292

[hbm70140-bib-0021] Himmelberg, M. M. , E. Tünçok , J. Gomez , K. Grill‐Spector , M. Carrasco , and J. Winawer . 2023. “Comparing Retinotopic Maps of Children and Adults Reveals a Late‐Stage Change in How V1 Samples the Visual Field.” Nature Communications 14, no. 1: 1561. 10.1038/s41467-023-37280-8. PMC10030632 36944643

[hbm70140-bib-0020] Himmelberg, M. M. , J. W. Kurzawski , N. C. Benson , D. G. Pelli , M. Carrasco , and J. Winawer . 2021. “Cross‐Dataset Reproducibility of Human Retinotopic Maps.” NeuroImage 244: 118609. 10.1016/j.neuroimage.2021.118609. 34582948 PMC8560578

[hbm70140-bib-0022] Horton, J. C. , and J. C. Hoyt . 1991. “The Representation of the Visual Field in Human Striate Cortex: A Revision of the Classic Holmes Map.” Archives of Ophthalmology 109, no. 6: 816. 10.1001/archopht.1991.01080060080030. 2043069

[hbm70140-bib-0023] Infanti, E. , and D. S. Schwarzkopf . 2020. “Mapping Sequences Can Bias Population Receptive Field Estimates.” NeuroImage 211: 116636. 10.1016/j.neuroimage.2020.116636. 32070751

[hbm70140-bib-0024] Jenkinson, M. , P. Bannister , M. Brady , and S. Smith . 2002. “Improved Optimization for the Robust and Accurate Linear Registration and Motion Correction of Brain Images.” NeuroImage 17, no. 2: 825–841. 10.1016/s1053-8119(02)91132-8. 12377157

[hbm70140-bib-0025] Keliris, G. A. , Q. Li , A. Papanikolaou , N. K. Logothetis , and S. M. Smirnakis . 2019. “Estimating Average Single‐Neuron Visual Receptive Field Sizes by fMRI.” Proceedings of the National Academy of Sciences 116, no. 13: 6425–6434. 10.1073/pnas.1809612116. PMC6442598 30867291

[hbm70140-bib-0026] Klein, A. , S. S. Ghosh , F. S. Bao , et al. 2017. “Mindboggling Morphometry of Human Brains.” PLoS Computational Biology Edited by D. Schneidman 13, no. 2: e1005350. 10.1371/journal.pcbi.1005350. 28231282 PMC5322885

[hbm70140-bib-0027] Lanczos, C. 1964. “A Precision Approximation of the Gamma Function.” Journal of the Society for Industrial and Applied Mathematics Series B Numerical Analysis 1, no. 1: 86–96. 10.1137/0701008.

[hbm70140-bib-0028] Larsson, J. , and D. J. Heeger . 2006. “Two Retinotopic Visual Areas in Human Lateral Occipital Cortex.” Journal of Neuroscience 26, no. 51: 13128–13142. 10.1523/JNEUROSCI.1657-06.2006. 17182764 PMC1904390

[hbm70140-bib-0030] Lerma‐Usabiaga, G. , M. Carreiras , and P. M. Paz‐Alonso . 2018. “Converging Evidence for Functional and Structural Segregation Within the Left Ventral Occipitotemporal Cortex in Reading.” Proceedings of the National Academy of Sciences 115, no. 42: E9981–E9990. 10.1073/pnas.1803003115. PMC6196482 30224475

[hbm70140-bib-0029] Lerma‐Usabiaga, G. , N. Benson , J. Winawer , and B. A. Wandell . 2020. “A Validation Framework for Neuroimaging Software: The Case of Population Receptive Fields.” PLoS Computational Biology Edited by S. Jbabdi 16, no. 6: e1007924. 10.1371/journal.pcbi.1007924. 32584808 PMC7343185

[hbm70140-bib-0031] Linhardt, D. , M. Pawloff , A. Hummer , et al. 2021. “Combining Stimulus Types for Improved Coverage in Population Receptive Field Mapping.” NeuroImage 238: 118240. 10.1016/j.neuroimage.2021.118240. 34116157

[hbm70140-bib-0032] Linhardt, D. , M. Pawloff , M. Woletz , et al. 2022. “Intrasession and Intersession Reproducibility of Artificial Scotoma pRF Mapping Results at Ultra‐High Fields.” vol. 9, no. 5. 10.1523/eneuro.0087-22.2022. PMC9512620 36635900

[hbm70140-bib-0033] Logothetis, N. K. 2008. “What We Can Do and What We Cannot Do With fMRI.” Nature 453, no. 7197: 869–878. 10.1038/nature06976. 18548064

[hbm70140-bib-0034] Moeller, S. , E. Yacoub , C. A. Olman , et al. 2010. “Multiband Multislice GE‐EPI at 7 Tesla, With 16‐Fold Acceleration Using Partial Parallel Imaging With Application to High Spatial and Temporal Whole‐Brain fMRI.” Magnetic Resonance in Medicine 63, no. 5: 1144–1153. 10.1002/mrm.22361. 20432285 PMC2906244

[hbm70140-bib-0035] Morgan, C. , and D. S. Schwarzkopf . 2020. “Comparison of Human Population Receptive Field Estimates Between Scanners and the Effect of Temporal Filtering.” F1000Research 8: 1681. 10.12688/f1000research.20496.2. PMC6913234 31885863

[hbm70140-bib-0036] Pawloff, M. , A. Hummer , M. Woletz , et al. 2019. “Retinotopic Mapping of the Primary Visual Cortex as an Objective Functional Adjunct to Conventional Testing in Patients With Retinal Disease.” Investigative Ophthalmology & Visual Science 60, no. 9: 4745.

[hbm70140-bib-0037] Prabhakaran, G. T. , K. O. al‐Nosairy , C. Tempelmann , H. Thieme , and M. B. Hoffmann . 2021. “Mapping Visual Field Defects With fMRI—Impact of Approach and Experimental Conditions.” Frontiers in Neuroscience 15, no. 1180: 745886. 10.3389/fnins.2021.745886. 34566575 PMC8455880

[hbm70140-bib-0038] Reuter, M. , H. D. Rosas , and B. Fischl . 2010. “Highly Accurate Inverse Consistent Registration: A Robust Approach.” NeuroImage 53, no. 4: 1181–1196. 10.1016/j.neuroimage.2010.07.020. 20637289 PMC2946852

[hbm70140-bib-0039] Ribeiro, F. L. , S. Bollmann , and A. M. Puckett . 2021. “Predicting the Retinotopic Organization of Human Visual Cortex From Anatomy Using Geometric Deep Learning.” NeuroImage 244: 118624. 10.1016/j.neuroimage.2021.118624. 34607019

[hbm70140-bib-0041] Schira, M. M. , C. W. Tyler , B. Spehar , and M. Breakspear . 2010. “Modeling Magnification and Anisotropy in the Primate Foveal Confluence.” PLoS Computational Biology Edited by G. Deco 6, no. 1: e1000651. 10.1371/journal.pcbi.1000651. 20126528 PMC2813258

[hbm70140-bib-0040] Schira, M. M. , C. W. Tyler , M. Breakspear , and B. Spehar . 2009. “The Foveal Confluence in Human Visual Cortex.” Journal of Neuroscience 29, no. 28: 9050–9058. 10.1523/JNEUROSCI.1760-09.2009. 19605642 PMC6665445

[hbm70140-bib-0042] Schwartz, E. L. 1977. “Spatial Mapping in the Primate Sensory Projection: Analytic Structure and Relevance to Perception.” Biological Cybernetics 25, no. 4: 181–194. 10.1007/BF01885636. 843541

[hbm70140-bib-0043] Schwartz, E. L. 1980. “Computational Anatomy and Functional Architecture of Striate Cortex: A Spatial Mapping Approach to Perceptual Coding.” Vision Research 20, no. 8: 645–669. 10.1016/0042-6989(80)90090-5. 7445436

[hbm70140-bib-0044] Silva, M. F. , J. W. Brascamp , S. Ferreira , M. Castelo‐Branco , S. O. Dumoulin , and B. M. Harvey . 2018. “Radial Asymmetries in Population Receptive Field Size and Cortical Magnification Factor in Early Visual Cortex.” NeuroImage 167: 41–52. 10.1016/j.neuroimage.2017.11.021. 29155078

[hbm70140-bib-0045] Strasburger, H. 2022. “On the Cortical Mapping Function—Visual Space, Cortical Space, and Crowding.” Vision Research 194: 107972. 10.1016/j.visres.2021.107972. 35182892

[hbm70140-bib-0046] Strasburger, H. , I. Rentschler , and M. Juttner . 2011. “Peripheral Vision and Pattern Recognition: A Review.” Journal of Vision 11, no. 5: 13. 10.1167/11.5.13. PMC11073400 22207654

[hbm70140-bib-0047] Tustison, N. J. , B. B. Avants , P. A. Cook , et al. 2010. “N4ITK: Improved N3 Bias Correction.” IEEE Transactions on Medical Imaging 29, no. 6: 1310–1320. 10.1109/TMI.2010.2046908. 20378467 PMC3071855

[hbm70140-bib-0048] Urale, P. W. B. , A. M. Puckett , A. York , D. Arnold , and D. S. Schwarzkopf . 2022. “Highly Accurate Retinotopic Maps of the Physiological Blind Spot in Human Visual Cortex.” Human Brain Mapping 43, no. 17: 5111–5125. 10.1002/hbm.25996. 35796159 PMC9812231

[hbm70140-bib-0049] van Dijk, J. A. , B. de Haas , C. Moutsiana , and D. S. Schwarzkopf . 2016. “Intersession Reliability of Population Receptive Field Estimates.” NeuroImage 143: 293–303. 10.1016/j.neuroimage.2016.09.013. 27620984 PMC5139984

[hbm70140-bib-0050] Wandell, B. A. , S. O. Dumoulin , and A. A. Brewer . 2007. “Visual Field Maps in Human Cortex.” Neuron 56, no. 2: 366–383. 10.1016/j.neuron.2007.10.012. 17964252

[hbm70140-bib-0052] Wang, L. , R. E. B. Mruczek , M. J. Arcaro , and S. Kastner . 2015. “Probabilistic Maps of Visual Topography in Human Cortex.” Cerebral Cortex 25, no. 10: 3911–3931. 10.1093/cercor/bhu277. 25452571 PMC4585523

[hbm70140-bib-0051] Zhang, Y. , M. Brady , and S. Smith . 2001. “Segmentation of Brain MR Images Through a Hidden Markov Random Field Model and the Expectation‐Maximization Algorithm.” IEEE Transactions on Medical Imaging 20, no. 1: 45–57. 10.1109/42.906424. 11293691

