# Supplementary figures and images for: Biases in Volumetric Versus Surface Analyses in Population Receptive Field Mapping
Source: Hum Brain Mapp. 2025 Jan 24;46(2):e70140. doi: 10.1002/hbm.70140 (PMC11758450; doi:10.1002/hbm.70140)

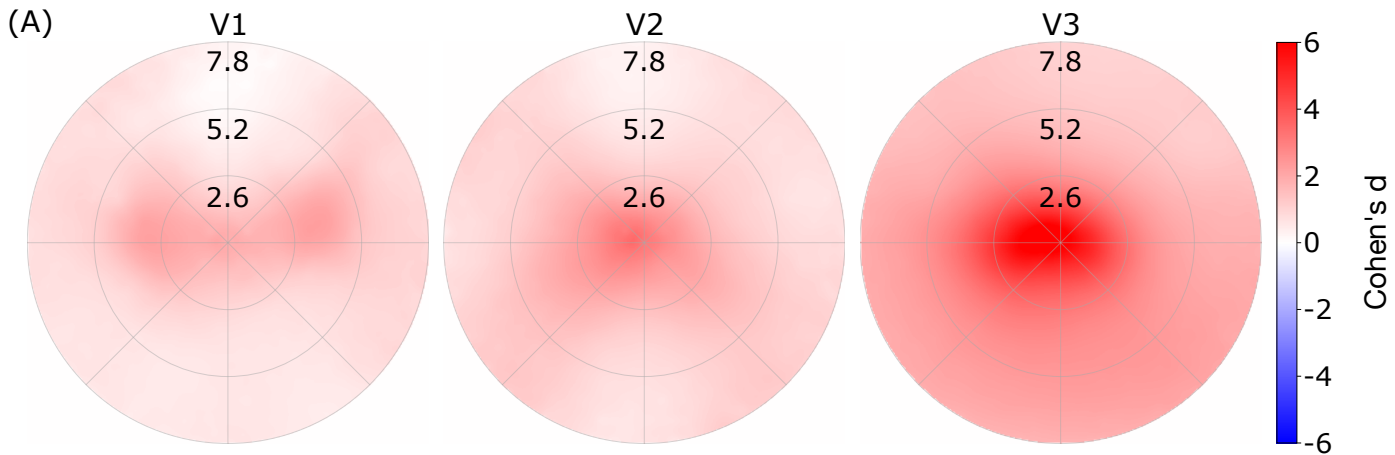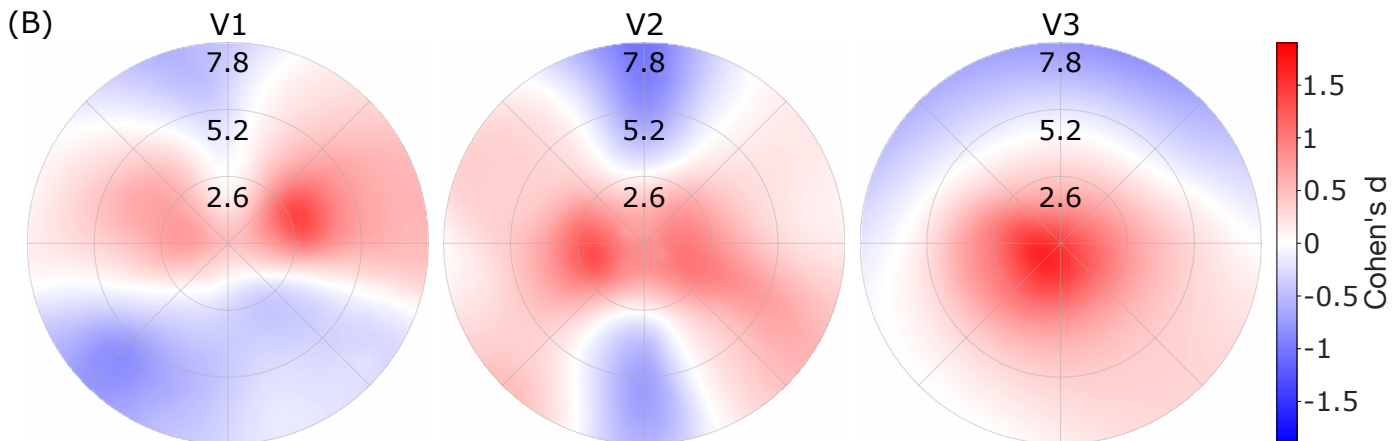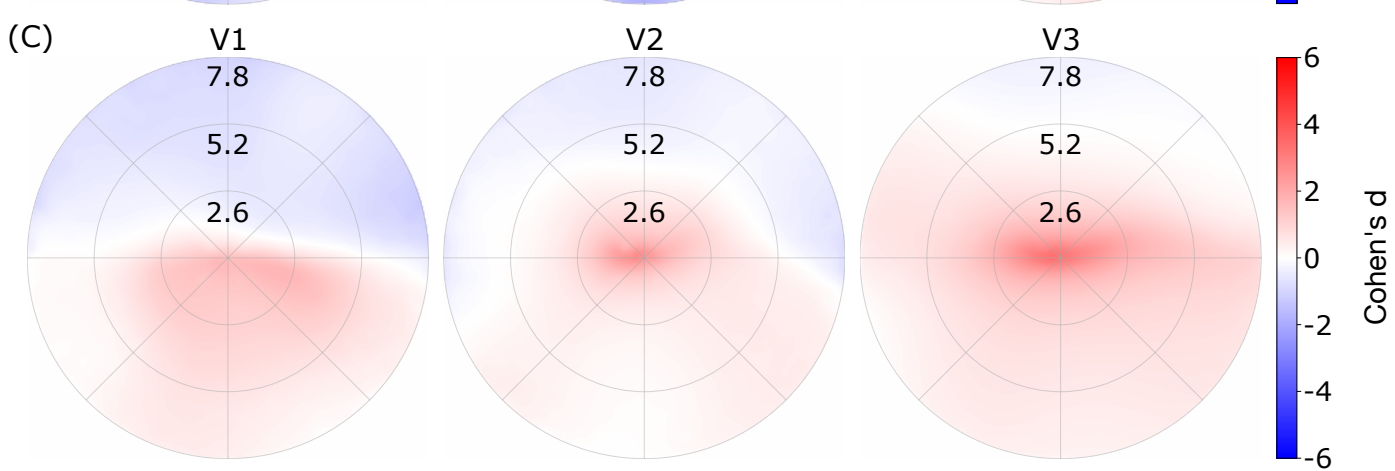

Supplement: Supplementary file 1 — Figure S1. Cohen’s d comparisons for V1‐3 and different analyses: A. Noiseless data analyzed projected to the surface using a nearest‐neighbor approach compared to volume analysis. The overall bias is slightly reduced compared to Figure 3A but remains present. B. Surface data thresholded at 41% variance explained compared to volume data with 20% threshold. The foveal bias is slightly increased compared to Figure 2B. C. Noiseless data were analyzed in the volume space with pRF parameters subsequently projected to the surface. This approach reduces the bias compared to projecting time courses to the surface and analyzing them there, as in Figure 3A. [file HBM-46-e70140-s001.pdf]
